# Different growth patterns in two siblings with Schimke immuno-osseous-dysplasia

**DOI:** 10.1007/s00467-024-06503-5

**Published:** 2024-09-18

**Authors:** Arend Bokenkamp, Antonia Bouts, Neeltje van der Weerd, Elena Levtchenko, Dieter Haffner, Miroslav Zivicnjak

**Affiliations:** 1 Department of Pediatric Nephrology, Emma Children’s Hospital, Meibergdreef 9, Amsterdam, NL-1105 AZ The Netherlands; 2 Department of Nephrology, Amsterdam University Medical Center, Amsterdam, The Netherlands; 3 Department of Pediatric Kidney, Liver and Metabolic Diseases, Hannover Medical School, Hannover, Germany

**Keywords:** Schimke immuno-osseous-dysplasia, Disproportionate short stature, Anthropometry

## Abstract

Schimke immuno-osseous-dysplasia (SIOD) is an autosomal recessive systemic disease due to pathogenic variants in *SMARCAL1*. Manifestations include nephrotic syndrome (NS), kidney failure, T-cell dysfunction, vaso-occlusive disease, and disproportionate short stature, a general feature of this disease. Here, we present a markedly different growth pattern in two brothers with SIOD sharing the same homozygous R561C missense variant. The index patient presented at the age of 11 years with NS and severely disproportionate short stature, followed by kidney failure at the age of 16, and severely reduced adult height (*z*-score − 8.0). In contrast, the younger brother showed normal growth until the age of 8 years. Mild proteinuria was noted at the age of 4.5, followed by NS at 9.5 years, kidney failure at 11 years, progressive disproportionate stature, and reduced adult height (*z*-score − 4.5). Both brothers had comparable disproportion in adulthood (sitting height index *z*-score − 0.88 and − 1.44, respectively).

## Case report

Schimke immuno-osseous-dysplasia (SIOD) is an autosomal recessive multi-system disease due to pathogenic variants in *SMARCAL1*, a gene involved in chromatin remodeling. Disease manifestations include nephrotic syndrome leading to kidney failure, T-cell dysfunction, and vaso-occlusive disease [1]. Disproportionate growth retardation is a hallmark of SIOD and is observed in 99% of patients, starting in utero in 70% of patients. Patients have a short neck and short trunk and increased lumbar scoliosis resulting in an abnormal sitting height/leg length ratio below 0.83, while patients with growth failure from chronic kidney failure have a ratio above 1.01 [2]. Here, we present long-term clinical and anthropometric data on two brothers with a R561C missense variant in *SMARCL1.*

The index patient presented at the age of 11 years with nephrotic syndrome [3]. On physical examination, he had the typical features of SIOD with severe short stature (height *z*-score − 4.36) and a low sitting height index (i.e., sitting height divided by total height) *z*-score of − 0.45. He had hyperpigmented maculae on the trunk, a triangular face with a depressed nasal bridge, and a broad nasal tip. The estimated glomerular filtration rate (eGFR) was 111 ml/min/1.73 m^2^. Treatment was symptomatic with an angiotensin-converting enzyme inhibitor (ACEi) as the nephrotic syndrome in SIOD is known to be resistant to steroids and other immunosuppressants. The patient developed kidney failure at the age of 16 years when he received a pre-emptive kidney transplant from a family donor. Apart from an early antibody-mediated rejection, the nephrological course was unremarkable; his estimated glomerular filtration rate (eGFR) at the age of 31 years is 41 ml/min/1.73 m^2^. He had a single transient ischemic attack at the age of 30 years and has not had any serious infections. Since the presentation, he only grew from 120 to 127 cm resulting in extreme stunting (height *z*-score − 8), while body proportions changed little (sitting height index *z*-score − 0.45 vs. − 1.23, respectively). At the last measurement, he had a high BMI of 33.8 kg/m^2^ and restrictive lung disease.

**Fig. 1:**
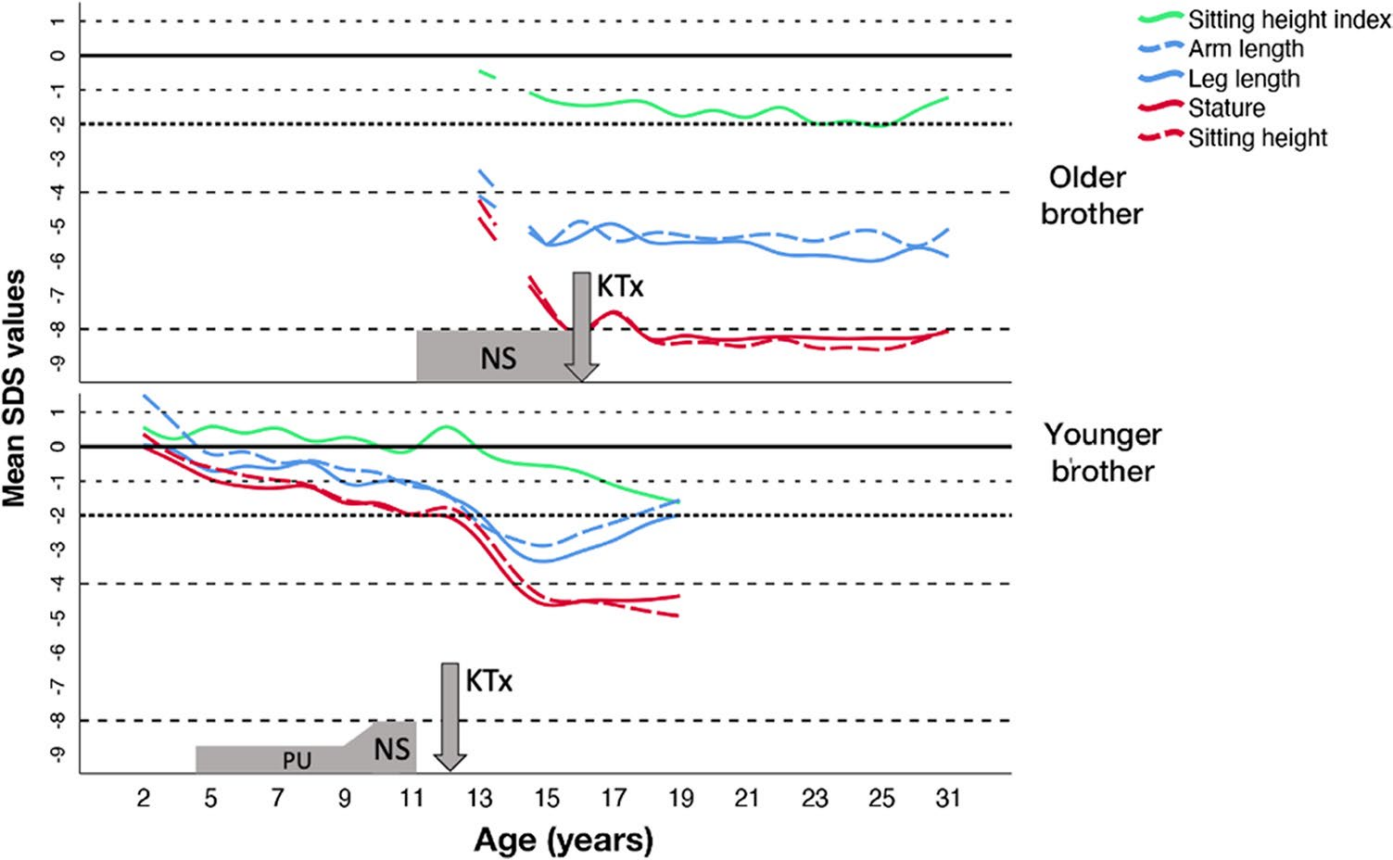
Growth patterns with respect to development of proteinuria and kidney transplantation. Detailed anthropometric measurements starting at the age of 13 and 3 years, respectively. Two missing measurements during the COVID-19 epidemic were interpolated (age 29 and 30 years in the older and 17 and 18 years in the younger brother). PU, proteinuria; NS, nephrotic syndrome; KTx, kidney transplantation

His younger brother was born at term with normal height, weight, and body proportions (Fig. 1). Apart from long eyelashes, he had no dysmorphic features or other clinical clues to the presence of SIOD. Mild proteinuria started at the age of 4.5 years and became nephrotic at the age of 9.5 years despite treatment with ACEi. He developed kidney failure at the age of 11 years and received a cadaveric kidney transplant at the age of 12 years which is functioning well 8 years after transplantation (eGFR 53 ml/min/1.73 m^2^). He has had no rejections, no serious infections, and no signs of vaso-occlusive disease.

The younger brother grew along the upper limit of his family’s target range (*z*-score 0 to − 3) until the age of 8 years when height velocity started to decline. The sitting height index *z*-score dropped below 0 only at the age of 13 years. His final height is 151 cm (*z*-score − 4.53) with a sitting height index *z*-score of − 1.44. Figure 1 illustrates that his final body proportions are comparable to his older brother, albeit with much higher *z*-scores.

In line with earlier series [1, 4], the present report illustrates the phenotypic variability of SIOD even in patients with an identical *SMARCL1* variant. Of note, four other patients with a missense variant in the R561 residue have been published, all of which had early onset short stature/intra-uterine growth retardation [5]. Due to the positive family history, the younger brother of the index patient could be evaluated prospectively from birth, which is a unique observation to the best of our knowledge. It took several years before mild proteinuria had turned into nephrotic syndrome. During this time, anthropometric data were within normal limits, and SIOD would not have been considered at first sight. Unlike his brother, he did not have hyperpigmented maculae or the facial features of SIOD. Therefore, without scrutinous analysis of serial measurements of different body dimensions (which are not available in clinical practice), it is likely that he would have been exposed to unsuccessful immunosuppressive treatment, and his underlying disease would only have been diagnosed by whole-exome sequencing. A potential clinical clue might have been the insidious start of proteinuria as opposed to the more rapid manifestation commonly seen in steroid-sensitive nephrotic syndrome. Of note, despite the early start of anti-proteinuric treatment, kidney failure developed much earlier and progressed much faster than in his older brother.

In conclusion, normal stature in a patient presenting with proteinuria does not rule out the presence of SIOD. The variability in the time course of kidney manifestations and severity of growth failure is yet unexplained.

## Summary

### What is new?

- SIOD can be missed clinically if the nephropathy manifests before the skeletal phenotype becomes evident. Proteinuria starts insidiously, and it may take years before overt nephrotic syndrome develops.
